# LLRHNet: Multiple Lesions Segmentation Using Local-Long Range Features

**DOI:** 10.3389/fninf.2022.859973

**Published:** 2022-05-05

**Authors:** Liangliang Liu, Ying Wang, Jing Chang, Pei Zhang, Gongbo Liang, Hui Zhang

**Affiliations:** ^1^College of Information and Management Science, Henan Agricultural University, Zhengzhou, China; ^2^Department of Computer Science, Eastern Kentucky University, Richmond, KY, United States

**Keywords:** iterative aggregation, transformer, image patches, long-range features, multiple lesions

## Abstract

The encoder-decoder-based deep convolutional neural networks (CNNs) have made great improvements in medical image segmentation tasks. However, due to the inherent locality of convolution, CNNs generally are demonstrated to have limitations in obtaining features across layers and long-range features from the medical image. In this study, we develop a local-long range hybrid features network (LLRHNet), which inherits the merits of the iterative aggregation mechanism and the transformer technology, as a medical image segmentation model. LLRHNet adopts encoder-decoder architecture as the backbone which iteratively aggregates the projection and up-sampling to fuse local low-high resolution features across isolated layers. The transformer adopts the multi-head self-attention mechanism to extract long-range features from the tokenized image patches and fuses these features with the local-range features extracted by down-sampling operation in the backbone network. These hybrid features are used to assist the cascaded up-sampling operations to local the position of the target tissues. LLRHNet is evaluated on two multiple lesions medical image data sets, including a public liver-related segmentation data set (3DIRCADb) and an in-house stroke and white matter hyperintensity (SWMH) segmentation data set. Experimental results denote that LLRHNet achieves state-of-the-art performance on both data sets.

## 1. Introduction

Deep convolutional neural networks (CNNs) have become the backbone of the development of artificial intelligence (Sarvamangala and Kulkarni, 2021). It is also becoming an essential prerequisite for segmenting medical images. Based on the CNN model, developing an automatic, accurate, and robust medical image segmentation model has become one of the hot issues in medical image analysis, it is the premise and foundation of diagnosis and image-guided surgery system. An accurate segmentation model can not only reduce the workload but also help clinicians improve work efficiency, make an accurate diagnosis and propose treatment strategies.

**Figure 1:**
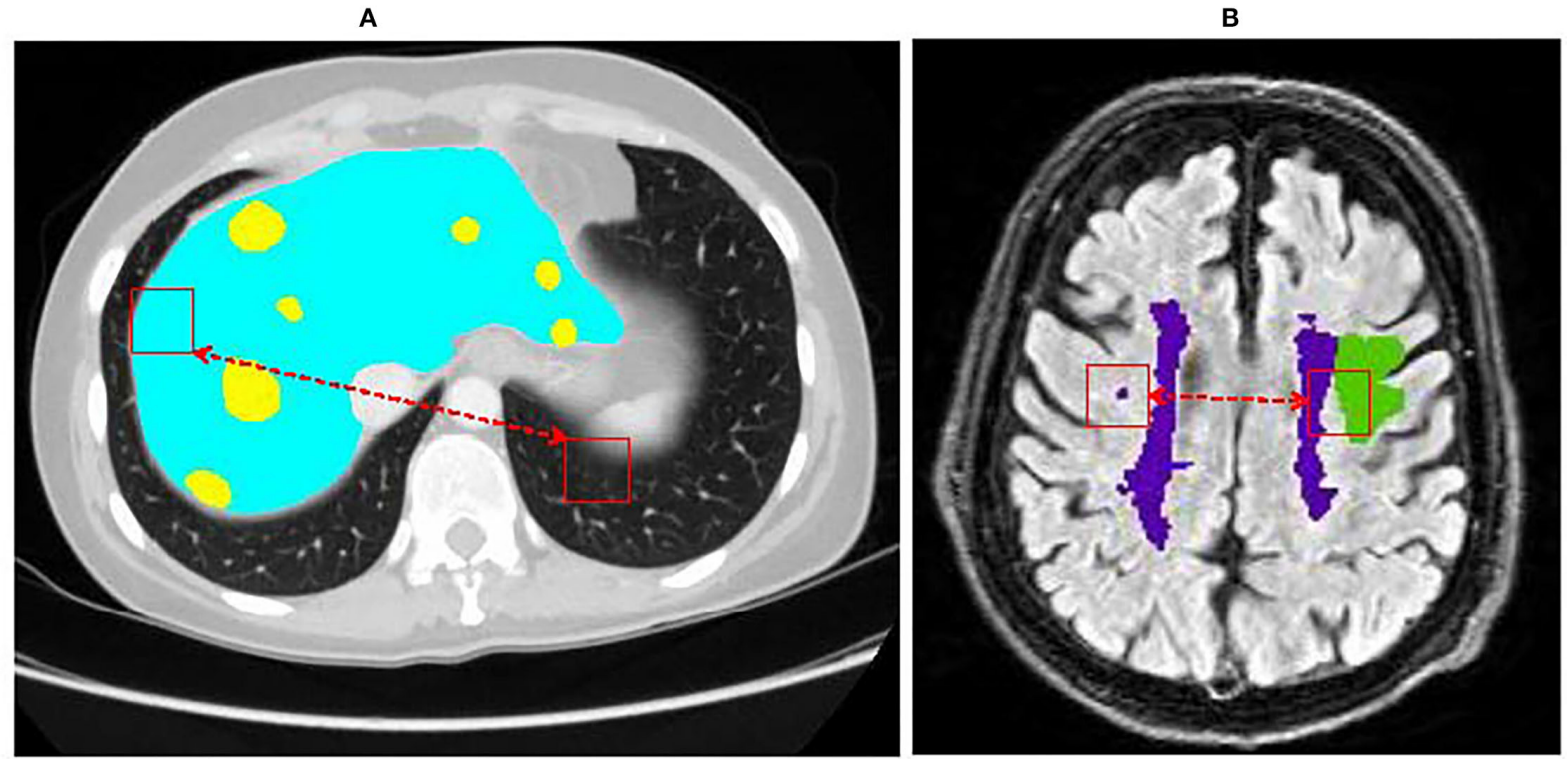
Examples of the multiple lesions medical images. **(A)** is liver and liver tumor lesions, **(B)** is stroke and white matter hyperintensity lesions.

In recent decade, deep learning methods have shown an adequate breakthrough in medical image segmentation tasks, which bring hope for the development of artificial intelligence in computer-aided diagnosis research (Bi et al., 2019). Some researchers have applied deep learning methods to multi lesion segmentation tasks (Li et al., 2013a; Christ et al., 2017; Hussain et al., 2018; Liu et al., 2020c). For example, Sun et al. (2017) proposed a fully convolutional network (FCN) for segmenting liver tumors. They designed a multi-channel fully convolutional network (MC-FCN) to segment liver tissues and tumors from multi-phase contrast-enhanced CT images. Hussain et al. (2018) proposed an automated glioma tumors segmentation DCNN. They used the patch-based manner to train the deep network by extracting two co-centric patches of different sizes from the input images. These studies have promoted the study of multiple lesion segmentation. However, the deep learning method still faces some great challenges in medical image segmentation tasks. (1) The boundaries of the lesion are quite similar, it's a challenge for CNNs to segment the boundaries. As shown in Figure 1, the original tissue in the color-labeled area is very similar to its surrounding tissue pixels, and it is difficult to distinguish. (2) It is difficult to establish a correlation between regions that are far apart. As shown in Figures 1A,B, it is difficult to mine out the relationship of the hidden pixel between the red blocks of long-distance convolution kernels. In recent years, with the improvement of computer hardware performance, deep learning methods have achieved impressive performance in the field of image segmentation, demonstrating the effectiveness of CNNs in learning discriminative features to segment organs or lesions from medical scans.

Convolutional neural networks are currently the basic building blocks of most methods proposed for image segmentation. In a CNN model, the local-range of convolution and the lost features in the down-sampling process, make the deep learning segmentation method different to obtain global feature information and make well-informed decisions. Although convolution operation can find the hidden association of pixels in different positions through the translation operation of convolution kernel, with the change of convolution kernel positions, the association among the pixels from the same lesion becomes more and more insignificant.

In order to alleviate the above problems, we propose a local-long range hybrid that features a deep CNN (LLRHNet) for multiple lesions segmentation, which consists of iterative aggregation and transformer technology. The main contributions are as follows:

1. We propose a local-long range hybrid features network for multiple lesions segmentation with the iterative aggregation mechanism and the transformer technology.
2. The iterative aggregation architecture learns the fusion of low and high-level local-range features from across layers.
3. The transformer technology adopts the multi-head self-attention mechanism to extract long-range features from image patches.
4. The local-long hybrid feature map helps LLRHNet reaches the advanced level on two multiple lesions medical image data sets.

The rest of this article is organized as follows. Section 2 shows the related studies. Sections 3 and 4 introduce the methodology and the material in our study. Some empirical comparative experiments are conducted in Section 5. Section 6 makes an extensive discussion about the LLRHNet network. Finally, Section 7 summarizes this study.

## 2. Related Studies

### 2.1. Semantic Segmentation

Semantic segmentation is an important component of computer vision. It is a natural step from rough reasoning to fine reasoning. Semantic segmentation refers to pixel-level image recognition, that is, marking the object category of each pixel in the image. Before deep learning methods are applied to the field of medical image analysis, researchers usually use TextonForest (Shotton et al., 2008) and random forest classifier (Maiora et al., 2014) as semantic segmentation tools. However, these semantic segmentation methods are difficult to achieve the rich representation of features from low level to a high level and resolutions from coarse to fine. CNN is developed recently. It can be used to analyze and mine data in a mechanism that is similar to the human brain. CNN is not only helpful for nature image analysis but also plays a great role in promoting the development of medical image semantic segmentation (Dora et al., 2017; Liu et al., 2020b).

### 2.2. Encoder-Decoder Models

To further mine the depth feature from the medical image, many researchers are devoted to the exploration of input data and network backbone. At first, patch-based deep learning methods are popular in semantic segmentation tasks (Xu et al., 2015; Volpi and Tuia, 2016), they used the image patch around the pixels to classify each pixel independently. Then, in 2014, Long et al. (2015) proposed an end-to-end FCN. FCN broke through the previous limitation that the patch-based method only used the fixed size of the input image so that CNN can carry out dense pixel prediction with the full connection layer. On this basis, Ronneberger et al. (2015) constructed a complete encoder-decoder model (U-net) in 2015. After that, almost all the advanced methods in the field of semantic segmentation adopt the encoder-decoder architecture as the backbone (Liu et al., 2020a; Nakarmi et al., 2020).

In a general encoder-decoder CNN model, the encoder network gradually reduces the high resolution of an image and extracts non-linear features. The decoder network projects the recognition feature (low resolution) semantics learned by the encoder into the pixel space (high resolution) to get a dense classification and gradually recover the location information. The encoder-decoder-based CNNs have shown the state-of-the-art performance in medical image segmentation tasks. For example, the U-shaped models were used in stroke and penumbra lesions segmentation (Liu et al., 2020c), white matter hyperintensity (WMH) lesions segmentation (Hongwei et al., 2018), liver and tumor segmentation (Li et al., 2018), skin cancer diagnosis (Andre et al., 2019), cardiac segmentation (Fu et al., 2018), histopathology image (van Rijthoven et al., 2021), and pancreas segmentation (Zhang et al., 2021b). However, there are two shortcomings in these CNNs: (1) This CNN focus on designing deeper or wider architectures but ignores the aggregate feature information across layers. (2) These CNNs cannot mine the long-range dependencies present in an image. More precisely, in a traditional encoder-decoder network, each convolutional kernel only focuses on the local-range pixels in an image rather than that across layers or the long-range. Therefore, it is worth paying attention to feature information in an aggregate manner and mining long-range dependent feature information, which will provide an accurate segmentation basis for the medical image segmentation method.

### 2.3. Transformer

The transformer is one of the extended mechanisms of attention CNN, which is proposed by Google in “Attention is all you need” (Vaswani et al., 2017). This model is widely used in natural language processing (NLP) applications (Devlin et al., 2018), such as machine translation, question answering system, text summarization, and speech recognition. Following their advantage in NLP applications, transformers have been adopted to image analysis tasks very recently (Touvron et al., 2021). Zheng et al. (2021) proposed a SEgmentation TRansformer (SETR) for nature image segmentation. They adopted a transformer as an encoder to transform the image into image patches and combined it with a decoder to make a powerful segmentation method. It is observed that these transformer-based methods can achieve the desired results on large-scale databases. In the field of medical image segmentation, the transformer-based method is in the ascendant. The closest studies are the ones that use attention mechanisms to boost the performance (Chen et al., 2021; Valanarasu et al., 2021). In particular, several studies in MICCAI2021 have achieved breakthroughs in medical image segmentation tasks by combining transformers with U-shaped networks (Wang et al., 2021; Zhang et al., 2021a). However, only using the transformer to encode the tokenized image patches, then directly sampling the hidden feature representation to obtain high-resolution dense output, and finally predicting segmentation, often can not produce satisfactory results.

## 3. Methodology

### 3.1. Overview of LLRHNet

**Figure 2:**
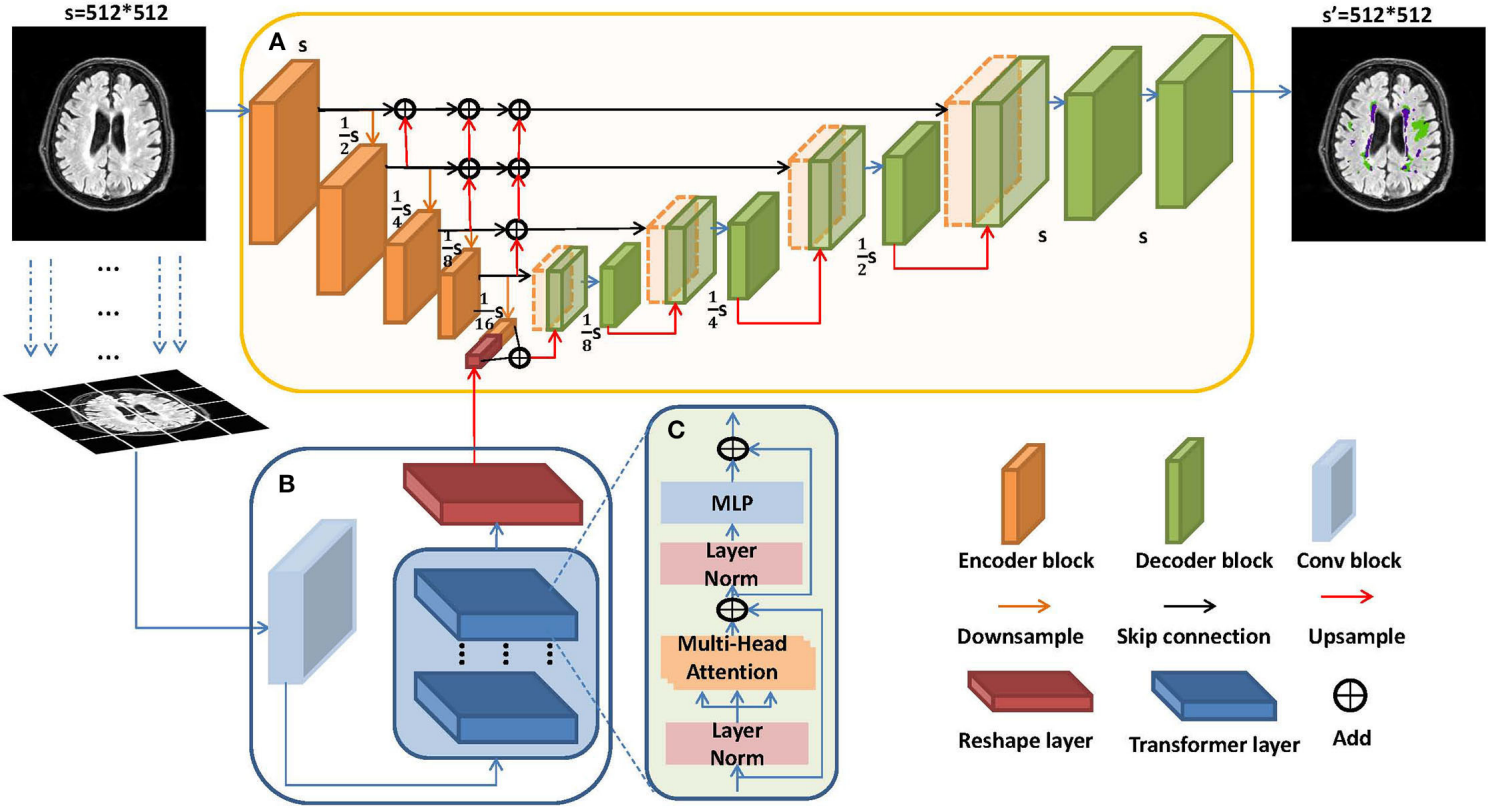
The architecture of local-long range hybrid features network (LLRHNet). **(A)** Local branch: the backbone of LLRHNet. **(B)** Global branch: the transformer block of LLRHNet. **(C)** The details of the transformer layer.

The architecture of LLRHNet is shown in Figure 2. LLRHNet has two branches: a local branch and a global branch. Figure 2A shows the encoder-decoder local branch which is inspired by the U-shaped architecture by Ronneberger et al. (2015). The U-shaped networks have shown adequate performance in medical image segmentation tasks (Liu et al., 2020b; Heller et al., 2021). In LLRHNet, the backbone of the local branch is based on ResNet (He et al., 2016). The local branch is used to extract the local-range features from a whole image. To achieve better cross-layer feature fusion, we use the multi-level feature iterative aggregation to replace the simple skip connection operation in the original ResNet. We iteratively aggregate different level features to learn a deep fusion of low and high-resolution features from isolated layers. Figure 2B shows the global branch which consists of an initial convolution layer, a reshape layer, and a transformer block. The transformer block is the main important element in the global branch. The main spirit of the transformer block is to extract the global/long-range feature from image patches. Figure 2C shows the details of the transformer layer. We use the transformer layers to learn the long-range pixel dependencies in an image. These layers emerge innate global multi-head attention mechanism that results in sufficient long-range details. We fuse the local-range features and the long-range features at the bottleneck layer of the local branch. It produces high-quality features for decoder layers, which in turn break the limited localization abilities due to insufficient local information from the local branch.

### 3.2. Network Architecture

We have outlined the architecture of LLRHNet in Section 3.1, we use LLRHNet to achieve two goals: (1) Using the global branch to obtain the long-range features from the images patches and combining the long-range features with the local-range information to assist the segmentation task. (2) Using the local branch to obtain local-range features from the whole image and complete the segmentation task. In this section, we will introduce the architecture of LLRHNet in detail.

#### 3.2.1. Iterative Aggregation Local Branch

The local branch adopts the encoder-decoder topology as the backbone, which is illustrated in Figure 2A. It is used to extract the local-range features from an image by the intrinsic locality in a convolution manner. The encoder-decoder topology serves as an outstanding performance network architecture in medical image analysis tasks (Chen et al., 2021). In our method, the encoder consists of 4 encoder blocks and 4 continuous down-sampling processes. The encoder block consisted of several convolution layers and ReLU, which is used to extract features. The down-sampling can convert the input image into a fixed-length vector. The decoder consists of 4 continuous up-sampling processes, which can convert the previously generated fixed vector into the prediction result. The skip connection operation is usually embedded into the encoder-decoder framework. It is used to introduce the low-level down-sampling features and concatenate with them in up-sampling layers, which is more conducive to generating a segmentation mask. This breaks the limitation that traditional skip connections are limited to cross-layers.

In the architecture of LLRHNet, we adopt the multi-level feature iterative aggregation skip connection replaces the traditional skip connection. We iteratively aggregate different level features to learn a deep fusion of low and high-resolution features. As shown in Figure 2A, we use the up-sampling operation to map the low-resolution features of the lower layer to that of the upper layer with the same resolution, and then we use the *add*() method to fuse the features of different layers. We obtain the low and high-level fusion features by using the iterative aggregation strategy. Our aggregation method realizes the feature fusion from shallow to deep among the isolated layers.

#### 3.2.2. Encoder of Global Branch

(1) Tokenized image patches

As shown in Figure 2B, the global branch is a shallow network. The transformer block takes the key component in this network. We hope the transformer block can extract long-range features from tokenized image patches. A standard transformer needs 1D sequences as input. Medical images consist of 2D slices. In our experiment, we need to convert the 2D slice into the 1D tokenized image patches.

Let a 2D image *X* with a spatial resolution of *H*×*W* and several channels of *C* ($X\in {\mathbb{R}}^{C_{in}\times H\times W}$). Finally, LLRHNet predicts the pixel-wise segmentation result $Y\in {\mathbb{R}}^{C_{out}\times H\times W}$. To handle 2D medical images, we draw on the experience of Dosovitskiy et al. (2020). We partition an input image into non-overlapping patches by convolution kernel in the first convolution block. Each image patch has a separate token. These tokens form an ordered sequence. The input image *X* is partitioned into several small 2D patches. Let the patch size of *P* × *P*, a flattened 2D image patch $x_{P}^{i}$ can be defined as follows:


(1) $$\begin{array}{l} x_{P}^{i}\in {\mathbb{R}}^{P^{2}.C_{in}}|\;i=1..N, \end{array}$$

where $N=\frac{HW}{P^{2}}$ is the number of patches in an image.

In our experiment, the patch size is *C*_*in*_ × 8 × 8. We use the convolution layer to embed the tokenized patches to the dimension of channel *C*_*in*_.

(2) Transformer

The transformer block is the main component of the global branch. Transformer models have been demonstrated exemplary performance on a broad range of machine translation and NLP tasks (Vaswani et al., 2017). The transformer model uses the self-attention mechanism instead of the RNN sequential structure, which makes the model parallel training and has global information. The attention mechanisms have been used to improve the performance of the medical image segmentation model in the closest studies (Jin et al., 2018; Liu et al., 2020c). In our experiment, the transformer is used to extract long-range features from image patches. The details of the transformer are illustrated in Figure 2C. The transformer consists of a shifted widow based on multi-head self-attention (MSA) and multi-layer perceptron (MLP) modules. Two layer-normalization (LN) operations are applied before the MSA and MLP models, respectively. Two residual connection operations are also applied after the MSA and MLP models. $x_{P}^{i}$ is the *i* − *th* tokenized image patch, we use token number and location to generate the sequence of image patches, and then use the sequence as the input of the transformer layer. The first input sequence (*x*_*seq*_) is defined as follows:


(2) $$\begin{array}{l} x_{seq}=\left[x_{p}^{1};x_{p}^{2};...;x_{p}^{N}\right], \end{array}$$

where *N* is the number of image patches.

Let $\tilde{x_{l}}$ be the output features of the MSA module, *x*_*l*_ be the output features of the MLP module, they can be defined as follows:


(3) $$\begin{array}{l} \tilde{x_{l}}=MSA\left(LN\left(x_{l-1}\right)\right)+x_{l-1}, \end{array}$$

(4) $$\begin{array}{l} x_{l}=MLP\left(LN\left({\tilde{x}}_{l}\right)\right)+\tilde{x}, \end{array}$$

where *LN*() denotes the layer normalization operation.

To keep the output feature vectors in the global branch and that of the bottleneck layer in the local branch has the same dimension, we add a reshape layer followed by the transformer block. The reshape layer only changes the dimension of input data, but the content remains unchanged.

(3) Multi-head attention

The attention mechanism is first introduced in a sequence-to-sequence task in 2014 by Bahdanau et al. (2014). The self-attention mechanism is one of the variants of attention mechanisms, which not only can reduce the dependence on external information, but also can capture the internal correlation of data or features. Based on these characteristics, the self-attention mechanism is developed as a context aggregation module to obtain context semantic information. It has achieved encouraging results in image segmentation and object detection tasks (Hu et al., 2019; Zhao et al., 2020).

In the image analysis task, the single-head self-attention aims at extracting the interaction relationship between all pixels by encoding each pixel in terms of the global contextual features. In order to capture the global context feature, single-head self-attention is defined by 3 learnable weight matrices: Queries ($W^{Q}\in {\mathbb{R}}^{n\times d_{q}}$), Keys ($W^{K}\in {\mathbb{R}}^{n\times d_{k}}$), and Values ($W^{V}\in {\mathbb{R}}^{n\times d_{v}}$). The first input patch sequence *x*_*seq*_ has been defined in Equation 2, *x*_*seq*_ is the first projected onto three weight matrices to get $Q=x_{seq}W^{Q}$, $K=x_{seq}W^{K}$, and $V=x_{seq}W^{V}$. The output *y* of a single-head self-attention can be defined as follows:


(5) $$\begin{array}{l} y=\text{softmax}\left(\frac{QK^{T}}{\sqrt{d_{q}}}\right)V, \end{array}$$

However, the limitation of single-head self-attention is that it only focuses on one specific location. We use the multi-head attention as the component of the proposed transformer in LLRHNet. Multi-head attention is one of the attention mechanisms (Vaswani et al., 2017) and can pay several independent parallel attention to different important locations at the same time. Specifically, in a multi-head attention mechanism, different random initialization mapping matrices can map the input vectors to different subspaces, which helps the model analyze the input sequence from different perspectives. Multi-head attention comprises multiple self-attention blocks (let *h* be the self-attention block number). Each block has its own set of learnable weight matrices ($W_{i}^{Q}$, $W_{i}^{K}$, and $W_{i}^{V}$), where *i* ∈ [1, *h* − 1]. Let *X* be an input image, we define the output of a *h* heads multi-head attention as follows:


(6) $$\begin{array}{l} Y_{out}=concat\left[y_{1},y_{2},...,y_{h-1}\right], \end{array}$$

where $Y_{out}\in {\mathbb{R}}^{n\times h.d_{v}}$, *concat*() denotes the concatenate operation, it is projected onto a weight matrix $W\in {\mathbb{R}}^{h.d_{v}\times d}$.

(4) Local-gobal branch hybrid as encoder

To improve the overall pixel relationship in an image, we fuse the feature maps which come from two encoders of two branches in the LLRHNet. Both feature maps should have the same size. The feature map of the local branch bottleneck in 2D form. While the output sequence vector of the transformer block is 1D form, we first reshape the size of the 1D vector ($\frac{HW}{P^{2}}$) to a 2D feature map with the size of $\frac{H}{P}\times \frac{W}{P}$, and then we use a convolution (1 × 1) to change the channel size of the reshaped feature map. Finally, we use the up-sampling operation to change the size of the feature map to $\frac{H}{8}\times \frac{W}{8}$, which has the same size as the bottleneck feature map of the local branch. We use the *add*() operation to fuse long-range and local-range feature maps. For segmentation purposes, the fused feature map is represented to full resolution (*H* × *W*) by cascaded up-sampling operations, which are used to predict the final segmentation result.

## 4. Evaluation Data Set

We conduct experiments on two multiple lesions segmentation data sets: the 3DIRCADb liver/liver tumor data set and the SWMH stroke/WMH data set.

### 4.1. 3DIRCADb Data Set

The 3DIRCADb is the abbreviation of the 3D-IRCADb-01 data set, which is a public liver and liver tumor segmentation data set (https://www.ircad.fr/research/computer/). 3DIRCADb data set offers a set of liver and liver tumor lesions on CT images (512 × 512). It consists of 20 samples (10 women and 10 men). All samples are anonymous. The CT images were performed on 3D scans. 75 % of samples were diagnosed with hepatic tumors. CT images consist of high various and complex organs in the abdominal cavity. The closeness and similarity of these organs increase the difficulty of the segmentation. Following (Qin et al., 2018; Wasserthal et al., 2018), all 3D CT volumes are converted in a slice-by-slice fashion and the predicted 2D slices are stacked together to reconstruct the 3D prediction for evaluation.

### 4.2. SWMH Data Set

The SWMH data set is a sub-set of a local hospital clinical data set. All samples in the SWMH data set were diagnosed with ischemic stroke disease at a local hospital between 2016 and 2018. All 26 samples are anonymous. All samples are between 20 and 50 years old. Each sample with both stroke and WMH lesions. We exclude the samples from adolescents and older adults because adolescents' brains are still developing and their brain structures are unstable. Common brain diseases in the elderly affect the segmentation result of the target lesion (Beumer et al., 2016). The MRI scans were performed on a Philips Achieve 3.0T MRI system with the following acquisition parameters: slice thickness was set to 6 mm, the field of view was set to 230 × 230 mm, field strength was set to 3.0T, matrix size was set to 230 × 230 × 18, slices were set to 18, slice spacing was set to 1.0–1.5 mm, repetition time was set to 23 ms, echo time was set to 87 ms, and pixel size in the *x* − *y* plane was set to 0.9 × 0.9 or 1.51 × 1.90 mm.

The MRIs in SWMH were stored in DICOM format with DWI, FLAIR, T1, and T2 modalities. These MRI images were preprocessed, including format conversion (DICOM to NIfFI), skull-stripped (Cox, 1996), re-coregistered MRI sequences to the DWI, corrected for intensity inhomogeneity due to B1 variations (Tustison et al., 2010). We transform all 3D MRIs into 2D image slices in the axial direction. Finally, we get 468 2D images. The gold standards of these images are based on DWI images, which are semi-manual annotated by two experienced radiologists. The semi-manual annotated process follows the STandards for ReportIng Vascular changes on nEuroimaging (STRIVE) (Wardlaw et al., 2013).

### 4.3. Evaluation Metrics

The performance of LLRHNet is assessed by Dice coefficient (DC) (Milletari et al., 2016), Hausdorff distance (HD) (Huttenlocher et al., 1993), Volumetric overlap error (VOE), Relative Volume Difference (RVD), and Average Symmetric Surface Distance (ASSD). Let *P* and *G* be the prediction result image and ground truth, respectively. DC is used to evaluate the proportion overlap of the target area between two images (*DC* ∈ [0, 1]), which is defined as follows:


(7) $$\begin{array}{l} DC\left(P,G\right)=\frac{2|P⋂G|}{\left|P|+|G\right|}, \end{array}$$

where *P* and *G* are the prediction image and ground truth, respectively. A larger DC value denotes a better segmentation result.

The HD is sensitive to outliers, it is defined as follows:


(8) $$\begin{array}{l} HD\left(P,G\right)=\max\left\{\underset{p\in P}{\max}\underset{g\in G}{\min}d\left(p,g\right),\underset{g\in G}{\max}\underset{p\in P}{\min}d\left(g,p\right)\right\}, \end{array}$$

where *d*(*p, g*) is the Euclidean distance between the pixels *p* and *g*.

The VOE is defined as follows:


(9) $$\begin{array}{l} VOE\left(P,G\right)=1-\frac{\left|P\cap G\right|}{\left|P\cup G\right|}. \end{array}$$

The RVD is an asymmetric measure defined as follows:


(10) $$\begin{array}{l} RVD\left(P,G\right)=\frac{\left|G|-|P\right|}{\left|P\right|}. \end{array}$$

The ASSD is defined as follows:


(11) $$\begin{array}{l} ASSD\left(P,G\right)=\frac{1}{2}\left(\frac{\sum_{p\in P}min_{g\in G}d\left(p,g\right)}{\left|P\right|}+\frac{\sum_{g\in G}min_{p\in P}d\left(g,p\right)}{\left|G\right|}\right). \end{array}$$

For HD, VOE, RVD, and ASSD measures, the smaller the value is, the better is the segmentation result.

## 5. Experiments and Results

In this section, we first compare the LLRHNet with other state-of-the-art methods on two data sets. Then, we extend several experiments for ablating the important elements of LLRHNet.

### 5.1. Implementation Details

We use the DC loss function (*L*_*dc*_) to optimize LLRHNet and train all comparison methods. The *L*_*dc*_ loss function is defined as follows:


(12) $$\begin{array}{l} L_{dc}\left(P,G\right)=1-\frac{2|P⋂G|}{\left|P|+|G\right|}=1-\frac{2\sum_{\left(ij\right)}^{N}p_{ij}g_{ij}+\varepsilon }{\sum_{\left(ij\right)}^{N}p_{ij}^{2}+\sum_{\left(ij\right)}^{N}g_{ij}^{2}+\varepsilon }, \end{array}$$

where *p*_*ij*_ and *g*_*ij*_ are the pixels in *P* and *G*, respectively. *N* is the total pixel number in an image. When there has no target pixel or only a few target pixels in *P* and *G*, which will make *L*_*dc*_ change greatly and lead to unstable training. In order to avoid this situation, we adopt ε to maintain numerical stability. In our experiments, we use Adam (Kingma and Ba, 2014) as the optimizer of *L*_*dc*_.

In these two data sets, the sample sizes in the 3DIRCADb data set are 512 × 512, the sample sizes in the SWMH data set are 224 × 224. We use the Skimage package (Van der Walt et al., 2014) to resize all images in the SWMH data set to 512 × 512. LLRHNet is implemented in Pytorch. To alleviate the problem of over-fitting problem in the training process, we adopt the early stopping strategy. The initial parameters of LLRHNet are set as follows: the epoch= 80, the mini-batch size = 3, the learning rate = 0.001, the drop-out rate = 0.3, and the random weight initialization. All experiments are implemented on the NVIDIA GeForce Titan X Pascal CUDA GPU processor.

### 5.2. Results on 3DIRCADb Data Set

According to the metrics provided by the 3DIRCADb data set, we use DC, ASSD, VOE, RVD, and HD to evaluate the performance of all comparison models. We verify the LLRHNet model on the 3DIRCADb data set. We choose two types of comparison methods: (1) The method which has excellent performance on the data set; (2) The state-the-of-arts segmentation method. Tables 1, 2 show the liver and liver tumor segment results on the 3DIRCADb data set, respectively. We reproduce H-DenseUNet (Li et al., 2018), MRFNet (Christ et al., 2017; Liu et al., 2020d), MedT (Valanarasu et al., 2021), and TransUNet (Chen et al., 2021) methods according to the codes provided by the authors, then train and predict these codes under the same conditions, and finally get the segmentation results. For the rest of the comparison methods, we use the results provided in the literature.

**Table 1:** The results of liver segmentation on the 3DIRCADb data set. The best results are shown in bold.

**Model**	**DC**	**VOE**	**RVD**	**ASSD**	**HD**
Li et al. (2015)	-	9.15 (±1.44)	-0.07 (±3.64)	1.55 (±0.39)	3.15 (±0.98)
Lu et al. (2017)	-	9.36 (±3.34)	0.97 (±3.26)	1.89 (±1.08)	4.15 (±3.16)
MRFNet (Liu et al., 2020d)	97.75 (±0.80)	3.31 (±0.95)	0.31 (±1.38)	0.32 (±0.16)	2.19 (±5.16)
H-DenseUNet (Li et al., 2018)	98.20 (±1.00)	3.57 (±1.66)	**0.01** (±0.02)	1.28 (±2.02)	3.58 (±6.58)
MedT (Valanarasu et al., 2021)	97.76 (±0.71)	2.61 (±0.86)	0.14 (±0.06)	1.03 (±1.69)	2.83 (±5.90)
TransUNet (Chen et al., 2021)	98.43 (±1.08)	**2.29** (±0.97)	**0.01** (±0.14)	1.48 (±1.98)	3.58 (±6.58)
LLRHNet	**98.64** (±0.92)	3.13 (±1.87)	**0.01** (±0.06)	**0.28** (±1.20)	**2.03** (±4.89)

**Table 2:** The results of tumor segmentation on the 3DIRCADb data set. The best results are shown in bold.

**Model**	**DC**	**VOE**	**RVD**	**ASSD**	**HD**
Li et al. (2013b)	-	14.40 (±5.30)	-8.10 (±2.10)	2.40 (±0.80)	2.90 (±0.70)
H-DenseUNet (Li et al., 2018)	93.70 (±2.00)	11.68 (±4.33)	**-0.01** (±0.05)	**0.58** (±0.46)	1.87 (±2.33)
MRFNet (Liu et al., 2020d)	94.81 (±4.20)	6.87 (±5.98)	0.07(±0.16)	0.82 (±0.64)	6.74 (±0.64)
MedT (Valanarasu et al., 2021)	94.99 (±2.43)	6.57 (±4.38)	0.56 (±0.30)	0.73 (±0.58)	4.20 (±1.03)
TransUNet (Chen et al., 2021)	**95.06** (±1.89)	6.09 (±3.97)	0.54 (±0.22)	0.69 (±0.74)	2.01(±0.89)
LLRHNet	**95.06** (±1.31)	**6.04** (±4.67)	0.43 (±0.12)	**0.58** (±0.66)	1.93 (±0.71)

LLRHNet achieves the mean DC is 98.64%, VOE is 3.13%, RVD is 0.01 mm, ASSD is 0.28 mm, HD is 2.03 mm on liver tissues segmentation, and the mean DC is 95.06%, VOE is 6.04%, RVD is 0.43 mm, ASSD is 0.58 mm, and HD is 1.93 mm on liver tumor lesions segmentation, respectively. Compared with the other 9 methods in the liver segmentation task, LLRHNet obtains the best scores of 3 out of 5 metrics. Compared with the other 11 methods in the liver tumor segmentation task, LLRHNet obtains the best scores in 2 out of 5 metrics. DC is the main metric in the segmentation task, we use a DC-based paired *t*-test as an additional analysis index to measure the performance of the model. In the liver segmentation task, we choose the top 5 DC scores of MedT, MRFNet, TransUNet, SpecTr, and H-DenseUNet as the paired method of LLRHNet, respectively. The *p* − *values* are 3E-09, 5E-10, 2E-09,2.3E-06, and 4E-08, respectively. In the liver tumor segmentation task, we choose the top 4 DC scores of MRFNet, MedT, TransUNet, and SpecTr as the paired method of LLRHNet, respectively. The *p* − *values* are 3.9E-07, 5.7E-08, 1.5E-10, and 7.5E-10, respectively. In conclusion, other comparison methods use the traditional convolution operation to obtain the local-range context information, while MedT, TransUNet, SpecTr, and LLRHNet introduce the iterative aggregation and transformer block, which help these methods to obtain more optimized local-range features and long-range features. All of these help to improve the competitiveness of these transformer-based methods. Compared with MedT, TransUNet, and SpecTr, our proposed LLRHNet achieves strong competitiveness, ranking first and second in DC value on the two test sets, respectively.

### 5.3. Results on SWMH Data Set

According to the experimental implementation details in Section 5.1, we compare LLRHNet with several other segmentation methods on the SWMH data set, including U-Net (Ronneberger et al., 2015), uResNet (Guerrero et al., 2017), FC-ResNet (Drozdzal et al., 2017), RA-UNet (Jin et al., 2018), MRFNet (Liu et al., 2020d), MedT (Valanarasu et al., 2021), and TransUNet (Chen et al., 2021). We adopt DWI and FLAIR MRIs as inputs. In these experiments, we are more concerned with the overlap degree between prediction results and ground truths and the outliers. Consequently, we conduct experiments to evaluate the performance of these comparison methods on DC and HD metrics. The comparison methods with the optimal parameters are described or released by the authors. The results are displayed in Table 3.

**Table 3:** The results for the 8 considered methods on the stroke and white matter hyperintensity (SWMH) data set. The best results are shown in bold.

**Methods**	**Ischemic stroke segmentation**		**WMHs segmentation**	
	**DC**	**HD**	**DC**	**HD**
U-Net (Ronneberger et al., 2015)	52.35 (±7.50)	6.02 (±5.32)	50.06 (±8.18)	7.06 (±6.01)
uResNet (Guerrero et al., 2017)	70.80 (±9.90)	3.25 (±1.92)	67.16 (±7.20)	2.97 (±1.97)
RA-UNet (Jin et al., 2018)	72.95 (±7.20)	3.16 (±1.99)	71.76 (±6.50)	2.67 (±1.28)
FC-ResNet (Drozdzal et al., 2017)	73.50 (±7.60)	3.08 (±1.94)	71.20 (±7.80)	2.63 (±1.38)
MRFNet (Liu et al., 2020d)	77.04 (±2.35)	2.94 (±1.31)	73.65 (±3.38)	2.47 (±1.04)
MedT (Valanarasu et al., 2021)	79.00 (±2.99)	3.01 (±1.20)	77.98 (±2.01)	2.48 (±1.10)
TransUNet (Chen et al., 2021)	79.06 (±2.76)	2.79 (±0.99)	**78.02** (±3.21)	2.38 (±1.99)
LLRHNet	**79.10** (±2.63)	**2.70** (±1.51)	**78.02** (±3.10)	**2.27** (±2.01)

All of these methods adopt the encoder-decoder structure as the backbone. Overall, the cascaded down-sampling\up-sampling operations and the skip connections can improve the utilization of features and alleviate the vanishing gradient problem. However, these methods ignore the further fusion of features and the extraction of long-range semantic information, except for the MedT, TransUNet, SpecTr, and LLRHNet. LLRHNet achieves the mean DC and HD of 79.10 and 78.02% and 2.70 and 2.27 mm in ischemic stroke and WMHs segmentation tasks, respectively. LLRHNet outperforms the other 5 non-transformer-based methods(U-Net, uResNet, FC-ResNet, RA-UNet, and MRFNet) by combining iterative aggregation and transformer block into the encoder-decoder backbone. This is mainly due to two reasons: (1) We use iterative aggregation to extract and optimize the local-range features from different layers. (2) We use the transformer block is to extract the long-range features from the image patches. The fused local-range and long-range features make LLRHNet obtain more comprehensive context information and improve the accurate prediction results. Compared with other transformer-based methods(MedT, TransUNet, and SpecTr), LLRHNet achieves the state-of-the-art in two similar lesions segmentation tasks.

### 5.4. Visualization Analysis

**Figure 3:**
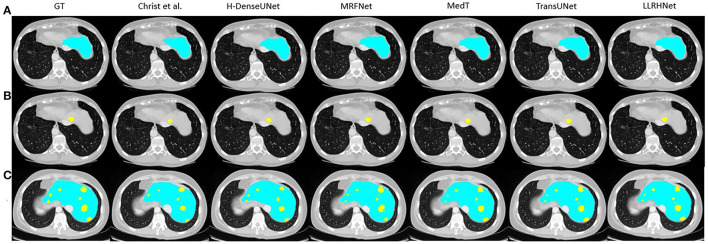
The visualization of 6 considered methods on the 3DIRCADb data set. The blue color represents the liver area and the yellow color represent the liver tumor tissue. **(A)** These images only contain liver tissue. **(B)** These images only contain tumor tissue. **(C)** These images contain both liver and tumor tissues.

**Figure 4:**
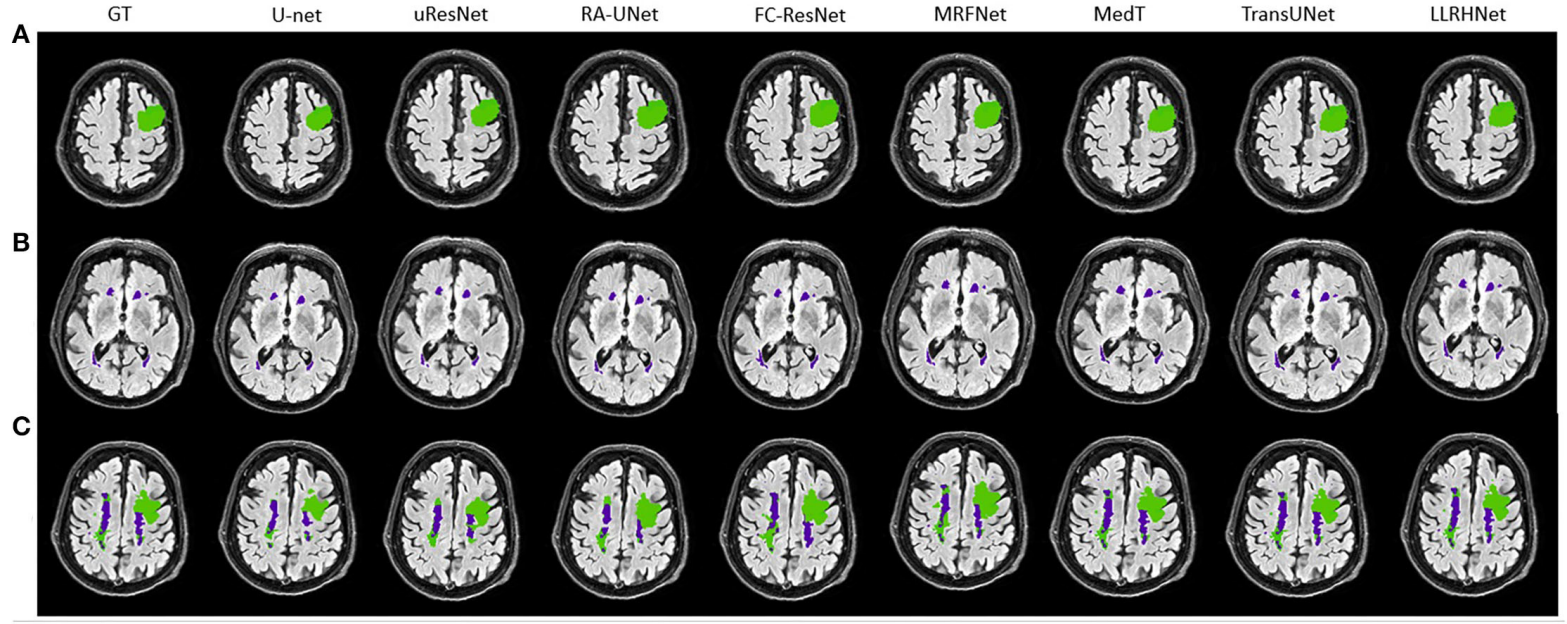
**(A–C)** The visualization of 8 considered methods on the stroke and white matter hyperintensity (SWMH) data set. The green color represents the stroke area and the purple color represents the WMH tissue.

To further intuitively analyze the performance of LLRHNet, we visualize several samples from two data sets. For the 3DIRCADb data set, we choose two samples as the visualization objects: one only contains liver tissue and another contains both liver tissue and tumor tissue. The visualization results are shown in Figure 3. For the SWMH data set, we select 3 samples as the visualization objects: one only contains stroke or WMH lesions and another contains both lesions at the same time. The visualization results are shown in Figure 4.

It can be found in Figures 3, 4 that the segmentation of single type tissue or lesion, the current advanced segmentation methods can accurately predict the range of target tissue, as shown in Figures 3A,B, 4A,B. The prediction results of LLRHNet and other methods are very close to the ground truths. Compared with other tissues, the correlations between pixels in the same type of tissue or lesion are relatively close. Convolution operation can find these correlations from the concerned local-range context information, and then make the correct pixel classification to predict the segmentation results. While for the samples with two types of target tissues at the same time, the prediction results of other non-transformer-based methods(U-Net, uResNet, FC-ResNet, RA-UNet, and MRFNet) and transformer-based methods(MedT, TransUNet, and SpecTr) are significantly different. As shown in Figures 3C, 4C, the prediction results of transformer-based methods are much closer to the ground truths. This is mainly caused by two reasons: (1) The pixels of two different types of target tissues interfere with each other, which limits the distinguishing ability of the model by the local-range property of convolution; (2) The iterative aggregation and transformer block are integrated into transformer-based methods. Transformer-based methods not only obtain the optimized local-range context information from across layers in the local branch but also integrates the long-range context information from the global branch so that transformer-based methods achieve the top performance in these methods. As shown in Tables 1–3, since the performance of these transformer-based methods is very close, their visualization graphs are highly similar, and it is difficult to tell the pros and cons directly from the visualization graphs.

## 6. Discussion

### 6.1. Ablation Experiments

#### 6.1.1. Influence of Patch Size

**Figure 5:**
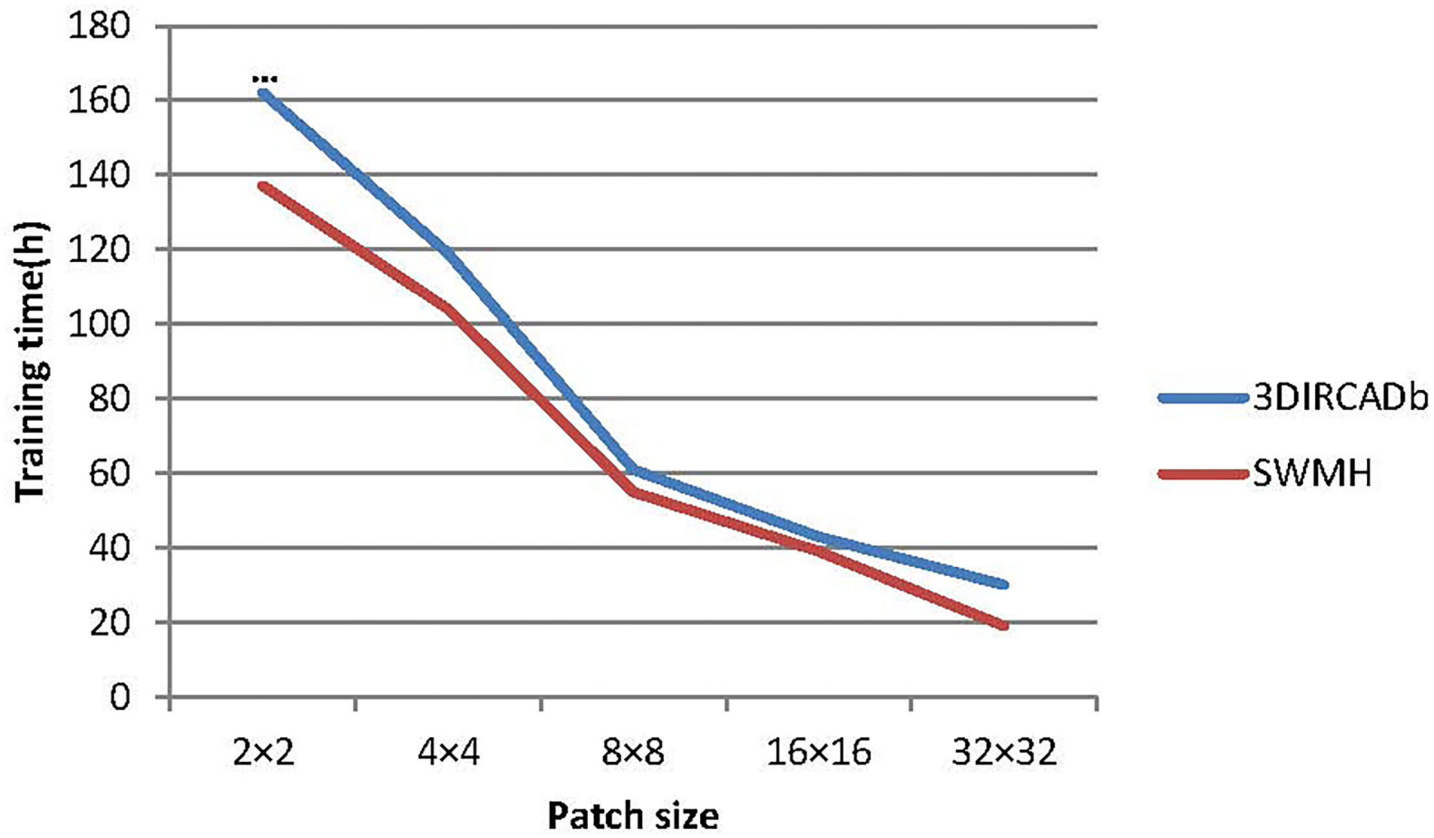
The time consuming of LLRHNet on different path size inputs.

**Figure 6:**
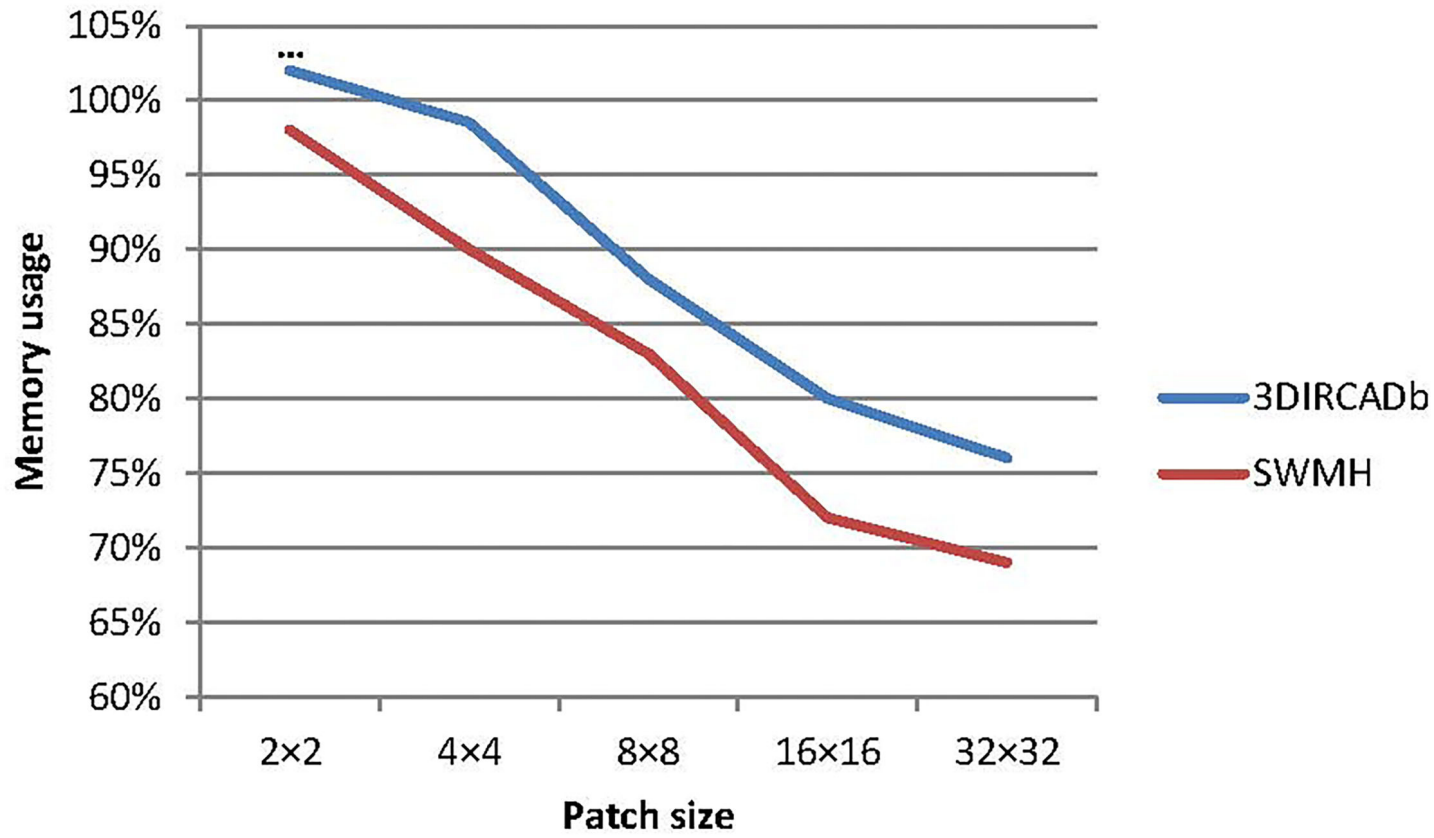
The memory consumption of LLRHNet on different path size inputs.

In our experiments, we finally use 8 × 8 as the size of the image patch in the transformer block. To verify the influence of patch size on the model performance, we choose 5 different image patch sizes (2 × 2, 4 × 4, 8 × 8, 16 × 16, and 32 × 32) to verify LLRHNet. The results are summarized in Table 4. At the same time, we compare the model training time and memory consumption of using different image patch sizes as global branch inputs, which are shown in Figures 5, 6.

**Table 4:** Influence of patch size on LLRHNet. The best results are shown in bold.

**Patch sizes**	**3DIRCADb data set**				**SWMH data set**			
	**Liver-DC**	**Liver-HD**	**Tumor-DC**	**Tumor-HD**	**Stroke-DC**	**Stroke-HD**	**WMH-DC**	**WMH-HD**
2 × 2	-	-	-	-	79.06	2.71	**78.89**	**2.21**
4 × 4	**98.70**	3.26	94.91	**6.00**	78.93	2.77	78.55	2.37
8 × 8	98.64	**3.13**	**95.06**	6.04	**79.10**	**2.70**	78.02	2.27
16 × 16	97.39	3.81	93.86	7.01	78.47	3.03	76.85	2.42
32 × 32	93.06	5.02	90.27	9.08	74.62	3.18	72.53	3.02

In general, with the increase of image patch size in the training process, the accuracy of LLRHNet on the two data sets is gradually reduced, the training time and memory consumption are both gradually reduced. Except that *patchsize* = 2 × 2, LLRHNet is trained on the 3DIRCADb data set, the required memory exceeds the upper limit of the server memory, resulting in LLRHNet cannot run and make prediction result on the 3DIRCADb data set. In addition, it is observed that a smaller image patch size usually helps the model achieve higher segmentation performance. However, it should note that the sequence length of the transformer is inversely proportional to the size of a patch, the smaller the patch size is, the higher the computational cost of the model. This is due to the smaller the patch size is, the longer the input sequence of the transformer needs to be encoded from the more complex dependencies between each patch and the higher the computational cost is. In our experiments, although the segmentation result of *patchsize* = 8 × 8 is slightly lower (worse) than that of *patchsize* = 4 × 4, however, the model training time and memory consumption of *patchsize* = 8 × 8 are much lower (better) than that of *patchsize* = 4 × 4. Hence, we choose 8 × 8 as the final image patch size of LLRHNet by balancing the computational cost and accuracy.

#### 6.1.2. Influence of Components

To further investigate the contribution of iterative aggregation and transformer components of LLRHNet, we conduct several ablation experiments based on 3DIRCADb and SWMH data sets. Table 5 summarizes the prediction results on two data sets. The results of the LLRHNet1 (ResNet) are the baseline for the ablation experiments. We investigate whether LLRHNet1 is combined with iterative aggregation or transformer block can improve the model performance. LLRHNet2 and LLRHNet3 are compared with LLRHNet1, which uses iterative aggregation operation or global branch in LLRHNet1, respectively. The performance of LLRHNet2 and LLRHNet3 is obviously better than that of LLRHNet1. Specifically, for LLRHNet2 and LLRHNet3, the DC values are improved by 2.85 and 2.78% for the liver segmentation, 3.14 and 2.26% for the liver tumor segmentation, 5.07 and 6.28% for the stroke segmentation, 6.88 and 8.15% for the WMH segmentation, respectively. For the 3DIRCADb data set, the DC values of LLRHNet2 are higher (better) than that of LLRHNet3, while for the SWMH data set, the result is the opposite. The LLRHNet4 architecture embeds the iterative aggregation into the skip connection of the LLRHNet1 and adds a transformer block as the global branch of the LLRHNet1. For LLRHNet4, the DC values achieve 98.64 and 95.06% for liver and liver tumor segmentation, 79.10 and 78.02% for stroke and WMH segmentation, respectively. Compared with LLRHNet2, LLRHNet4 extends the transformer block as an assistant branch to extract the long-range features from image patches. In the bottleneck layer of the local branch, the local-range and the long-range features are fused, which improves the accuracy of segmentation results. Compared with LLRHNet3, LLRHNet4 keeps the global branch and embeds the iterative aggregation into the skip connection of the local branch, iterative aggregation can fuse low-high resolution among different layers which provides abundant information for LLRHNet4. Compared with other models, the ultimate architecture of LLRHNet4 obtains the top DC values on both data sets. This is due to the LLRHNet4 inheriting the high-quality local-range features and long-range features from the iterative aggregation and transformer block. In our experiments, we adopt LLRHNet4 as the final model. As shown in Table 5, the use of the iterative aggregation and transformer block help to improve the network to achieve higher performance.

**Table 5:** Predictive performance of different network architectures, with the mean values listed.

**Methods**	**3DIRCADb**					**SWMH**	
	**Liver and tumor**					**Stroke and WMH**	
	**DC**	**VOE**	**RVD**	**ASSD**	**HD**	**DC**	**HD**
LLRHNet1	93.51 & 89.17	6.01 & 12.37	0.81 & 7.64	1.57 & 4.16	1.21 & 4.72	69.76 & 65.31	3.89 & 4.61
LLRHNet2	96.36 & 92.31	4.97 & 9.26	0.80 & 3.08	1.05 & 2.76	1.34 & 4.37	74.83 & 72.19	3.87 & 3.08
LLRHNet3	96.29 & 91.43	5.30 & 8.86	0.91 & 4.36	0.97 & 2.43	2.37 & 3.54	75.04 & 73.47	3.41 & 2.98
LLRHNet4	**98.64 & 95.06**	**3.13 & 6.04**	**0.01 & 0.43**	**0.28 & 0.58**	**2.03 & 0.71**	**79.10 & 78.02**	**2.70 & 2.27**

*LLRHNet1 denotes ResNet, LLRHNet2 denotes ResNet only with iterative aggregation, LLRHNet3 denotes ResNet only with transformer, and LLRHNet4 is the final model we choose. The best results are shown in bold. DC:%, HD:mm, VOE:%, RVD:%, HD:mm*.

### 6.2. Visualization of the Local-Long Hybrid Feature Map

**Figure 7:**
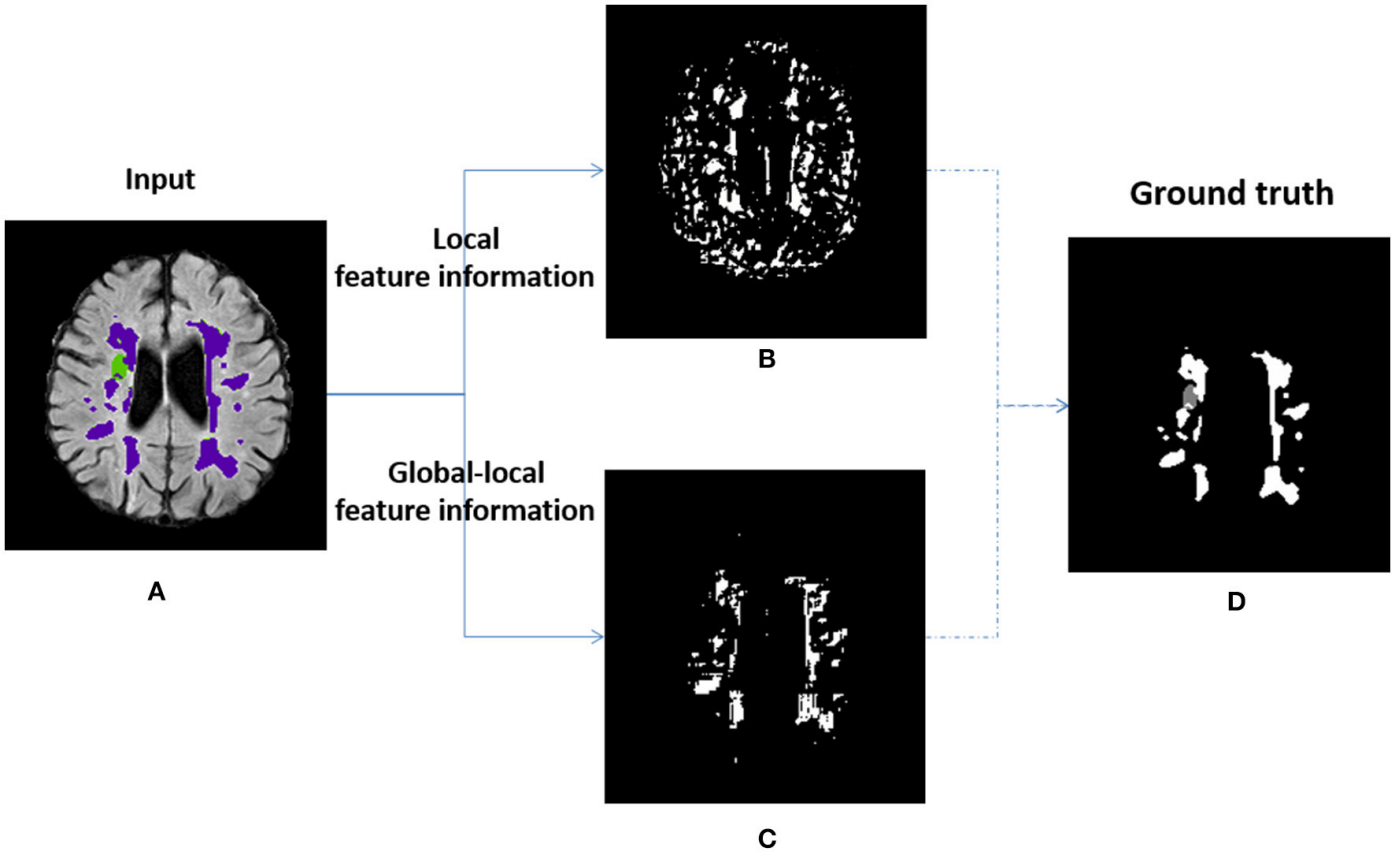
Visualization of the output layer bottleneck in the local branch of LLRHNet. **(A)** The input image. **(B)** The visualization of the local-feature. **(C)** The visualization of the global-local feature. **(D)** The ground truth.

The local-range features are obtained from the bottleneck layer in the local branch, the long-range features are obtained from the global branch, the long-range features strategy is the key assisted component to improve the richness of pixel context information for LLRHNet. As shown in Figure 2A, we use the *add*() method to fuse the local and long-range features. It ensures that LLRHNet can effectively obtain a hybrid feature map from local-range features and long-range features. This fusion operation produces a local-long hybrid feature map for the decoder layers. As shown in Figure 7, we output the intermediate layers that come from the scenario where the bottleneck layer is implemented with the long-range features in Figure 7C and without it in Figure 7B. Figures 7A,D show an input image and the ground truth, respectively.

In Figure 7B, we observe that when the long-range features are absent, the representation only comes from the local-range feature that has too many disturbing pixels, there is a lot of noise, and disagree with the ground truth (Figure 7D). In contrast to the above practice, we fuse the local-long features as a hybrid feature map. In Figure 7C, the long-range feature helps the decoder produce a rough prediction, however, it is much more similar to the ground truth. It denotes that the long-range features improve the ability of the decoder to recognize noisy pixels and optimize the segmentation results. This is mainly due to the fact that the transformer can mine the relationship between long-range pixels. The fusion of transformer block produces higher-quality intermediate feature information that has a better chance to converge into a high-quality prediction. As shown in Figures 7B–D, we note that only one kind of lesion tissue is identified, which indicates that the fused features have limitations on multi-target recognition. Improving the ability of hidden layers to recognize multi-target lesions is the issue we plan to study in the future.

### 6.3. Limitations

Although our approach achieves the best results in the automatic segmentation of multiple lesions, there are still some limitations in this study. First, the sample sizes of the two data sets we used were small, which limited the model to learning deep-level and discriminative features. Due to various conditions, it is difficult to collect data from multiple centers that meet the requirements. We intend to collect multi-center data in the future. Second, the transformer block is introduced into the LLRHNet. While this strategy improves model performance, it also leads to an increase in training parameters and training time. We choose U-Net, uResNet, MedT, and TransUNet as the typical representative for comparison. We compare the training time and parameters of these methods on the SWMH data set. U-Net and uResNet are shallow neural networks. MedT, TransUNet, and LLRHNet are transformer-based neural networks.

The results are shown in Table 6. It can be found that MedT and TransUNet, LLRHNet require more training time than U-Net and uResNet methods, which is mainly caused by the increase in the number of parameters after the introduction of the transformer block. Additionally, MedT, TransUNet, and LLRHNet all use the transformer technology, but compared with MedT and TransUNet, LLRHNet has advantages in both training time and the number of parameters. This is mainly because we use the U-shaped network as the main frame, and the transformer blocks are only used in the skip connection layers, which puts less burden on the model. From Table 6, it can be seen that the training time and parameters of LLRHNet have advantages over MedT and TransUNet. But the complexity of the LLRHNet still requires a lot of parameters and training time. Therefore, in the future study, we will focus on the problem of model optimization.

**Table 6:** The results of training time and parameters of U-Net, uResNet, TransUNet, and LLRHNet on SWMH data set.

**Model**	**Training time (hours)**	**Parameters**
U-Net	8.5	13M
uResNet	12	19M
MedT	28	33M
TransUNet	30	37M
LLRHNet	27.5	30M

## 7. Conclusion

We provide a deep learning network with iterative aggregation and transformer technology, called LLRHNet. LLRHNet can concurrently and accurately segment multiple lesions from medical images. The key architectural feature of LLRHNet is that it merits both iterative aggregation and transformer on the encoder-decoder backbone. The encoder-decoder backbone achieves local-range features extraction and targets location. The iterative aggregation can fuse the low and high-level local-range features from across layers. The transformer technology adopts the multi-head self-attention mechanism to extract long-range features from the tokenized image patches. LLRHNet is evaluated on two medical image data sets. Empirical comparison with well-established methods demonstrates that LLRHNet achieves competitive segmentation performance. Furthermore, we exhibit the ablation experiments and the representations of the bottleneck layer that explain the role of key components in our network. In the future study, we will pay attention to improving the ability of hidden layers to recognize multi-target lesions.

## Data Availability Statement

The original contributions presented in the study are included in the article/supplementary material, further inquiries can be directed to the corresponding author.

## Author Contributions

LL and HZ conceived and designed the study. JC, YW, and PZ performed the experiments. LL, YW, and GL reviewed and edited the manuscript. All authors read and approved the manuscript.

## Funding

The study described in this article was supported by the National Natural Science Foundation of China under Grant Nos. 61772557, 61772552, 61622213, and 61728211. The Foundation of Henan Agricultural University No. 2022-XGDC-02, and the Henan Provincial Key Research and Promotion Projects (No. 222102310085).

## Conflict of Interest

The authors declare that the research was conducted in the absence of any commercial or financial relationships that could be construed as a potential conflict of interest.

## Publisher's Note

All claims expressed in this article are solely those of the authors and do not necessarily represent those of their affiliated organizations, or those of the publisher, the editors and the reviewers. Any product that may be evaluated in this article, or claim that may be made by its manufacturer, is not guaranteed or endorsed by the publisher.
